# Facile Synthesis of Self-Adhesion and Ion-Conducting 2-Acrylamido-2-Methylpropane Sulfonic Acid/Tannic Acid Hydrogels Using Electron Beam Irradiation

**DOI:** 10.3390/polym15183836

**Published:** 2023-09-20

**Authors:** Hee-Woong Park, Nam-Gyu Jang, Hyun-Su Seo, Kiok Kwon, Seunghan Shin

**Affiliations:** 1 Green Chemistry & Materials Group, Korea Institute of Industrial Technology (KITECH), Cheonan 31056, Republic of Korea; binufamily@kitech.re.kr (H.-W.P.); cnoo7546@kitech.re.kr (N.-G.J.); syuiopa77@gmail.com (H.-S.S.); kioks@kitech.re.kr (K.K.); 2 Department of Convergence Manufacturing System Engineering, University of Science & Technology (UST), Daejeon 34113, Republic of Korea

**Keywords:** AMPS, hydrogel, tannic acid, E-beam, peel strength

## Abstract

Tannic acid (TA) can be used as an additive to improve the properties of hydrogels, but it acts as a radical scavenger, which hinders radical polymerization. In this study, we successfully and easily synthesized a TA-incorporated 2-acrylamido-2-methylpropanesulfonic acid (AMPS) hydrogel using an electron beam (E-beam) in a one-pot process at room temperature. TA successfully grafted onto AMPS polymer chains under E-beam irradiation, but higher TA content reduced grafting efficiency and prevented hydrogel formation. Peel strength of the AMPS hydrogel increased proportionally with TA, but cohesive failure and substrate residue occurred above 1.25 phm (parts per 100 g of AMPS) TA. Tensile strength peaked at 0.25 phm TA but decreased below the control value at 1.25 phm. Tensile elongation exceeded 2000% with TA addition. Peel strength varied significantly with substrate type. The wood substrate had the highest peel strength value of 150 N/m, while pork skin had a low value of 11.5 N/m. However, the addition of TA increased the peel strength by over 300%. The ionic conductivity of the AMPS/TA hydrogel increased from 0.9 S/m to 1.52 S/m with TA content, while the swelling ratio decreased by 50% upon TA addition and increased slightly thereafter.

## 1. Introduction

Tannic acid (TA) is a natural polyphenolic compound derived from plants, known for its water solubility. It possesses various properties such as biodegradability, thermal stability, antioxidant activity, and antibacterial properties [1,2]. In addition, there are cases where TA promotes the angiogenesis process within implanted scaffolds due to its biocompatible properties [3]. The presence of multiple phenolic hydroxyl groups in TA enables specific interactions with both organic and inorganic substances through hydrogen, ionic, and coordination bonds [4,5]. Therefore, researchers have explored the incorporation of TA into hydrogels to facilitate the formation of secondary forces with the substrates, thereby improving the adhesion and cohesion of the hydrogel [6,7,8].

However, the radical scavenging effect of TA, attributed to its antioxidant properties, has posed challenges in conducting radical polymerization using compositions containing TA. As a result, conventional approaches have primarily employed post-preparation soaking methods to introduce TA into hydrogels. For example, Meng et al. employed a photocuring technique followed by immersion in TA solution to improve mechanical properties of polylysine-based hydrogels [9]. Similarly, Fan et al. achieved enhanced adhesion and cohesion of polymer-based hydrogels through immersion in TA solution [8]. Wen et al. synthesized a gel based on polyethylene glycol-based crosslinked polyurethane, which was subsequently soaked in TA solution, resulting in significant improvements in adhesion and mechanical properties [10]. Nonetheless, these methods require additional soaking steps, leading to longer manufacturing times and increased complexity.

In a previous copolymerization study conducted by Bakhtawara et al., hydrogels with improved adhesion and mechanical properties were successfully prepared. This was achieved by adding an ammonium persulfate initiator to a solution containing 2-acrylamide-2-methylpropanesulfonic acid (AMPS) and TA, followed by stirring at 40 °C for more than 10 h [11]. Similarly, Cui et al. reported the grafting of TA onto the AMPS chain using a peroxide-based initiator at 75 °C for 3.5 h [12]. However, these thermal radical polymerization approaches suffer from drawbacks such as high temperature requirements and long reaction times [13].

An alternative method employing UV polymerization instead of thermal radical polymerization has been explored, offering a simpler and faster reaction at room temperature. However, the reactivity of radicals generated by UV irradiation is limited in aqueous solution phases, making it challenging to prepare hydrogels under typical UV irradiation conditions. Specifically, the radical scavenging effect of TA hinders the preparation of AMPS/TA hydrogels under atmospheric polymerization conditions [14,15]. Therefore, there is a demand for a polymerization technology capable of generating a large number of radicals that surpasses the radical scavenging effect of TA. E-beam radical polymerization emerges as a promising solution, as it does not require initiators or stabilizers, occurs rapidly at room temperature, and generates a significant amount of hydroxyl radicals when irradiating aqueous hydrogel solutions [16,17]. In addition, exposure to E-beam irradiation results in a surface modification that makes the surface more hydrophilic, which in turn improves adhesion properties and produces other beneficial effects [18].

AMPS-based hydrogels possess sulfonic acid groups and amide groups that are resistant to hydrolysis. These functional groups not only facilitate the formation of inter/intramolecular hydrogen bonds but also contribute to the high ionic conductivity exhibited by these hydrogels. As a result, AMPS-based hydrogels have the potential for extensive applications in skin contact electrodes, medical devices, drug delivery systems, and more [19,20]. However, the conventional method for preparing AMPS-based hydrogels primarily relies on thermal curing, with limited studies exploring the use of E-beam polymerization [13,21].

Therefore, in this study, we aimed to develop a relatively simple approach for preparing AMPS/TA hydrogels by irradiating AMPS/TA aqueous solutions with E-beam radiation. We prepared hydrogels with varying TA contents and observed changes in the hydrogel structure, gel fraction, and mechanical properties. Specifically, we focused on analyzing the mechanical properties, pore structure, water absorption, and ionic conductivity of the resulting hydrogels. Furthermore, we evaluated the adhesion properties of AMPS/TA hydrogels on different substrates. The AMPS/TA hydrogel prepared by E-beam irradiation promises to be a versatile solution. It can serve as an attachment pad for wearable devices such as electrical muscle simulators and electrocardiogram monitors, as well as a strain sensor.

## 2. Materials and Methods

### 2.1. Materials

**Figure 1:**
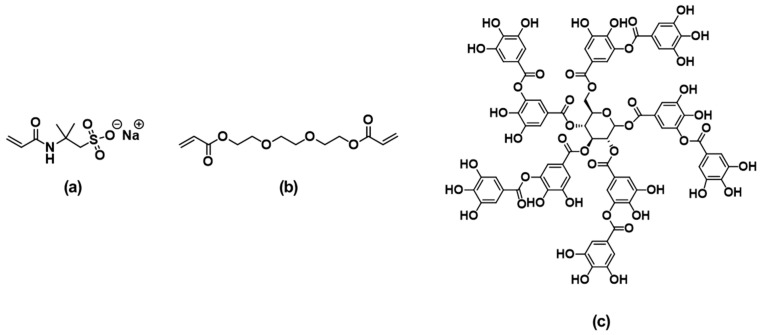
Structural formulas of chemicals used in hydrogel preparation. (**a**) AMPS (**b**) PEGDA (**c**) TA.

2-Acrylamido-2-methylpropanesulfonic acid sodium salt solution (AMPS, 50 wt% in H_2_O) was used as main monomer, polyethylene glycol diacrylate (PEGDA, Mn = 250) was used as a crosslinker, and tannic acid (TA, ACS reagent grade) was used as an additive. All reagents used in the experiment were purchased from Aldrich (St. Louis, MO, USA) and used without purification. Figure 1 shows the structures of the chemicals used in this study.

### 2.2. Synthesis of AMPS/TA Hydrogel by E-Beam Irradiation

**Figure 2:**
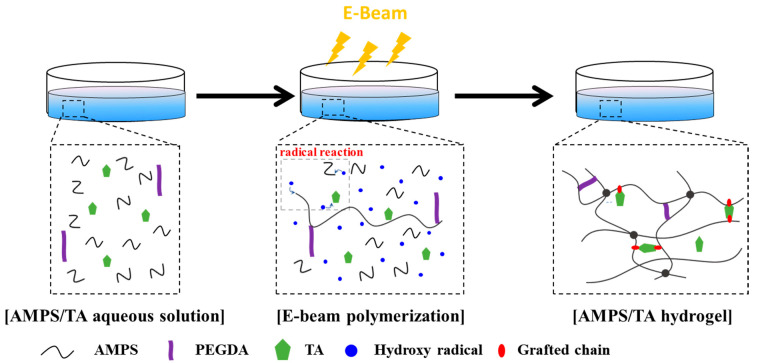
Synthesis of AMPS/TA hydrogels by E-beam irradiation.

AMPS/TA aqueous solutions were prepared according to the compositions shown in Table 1. For experiments involving grafting, gel fraction, and peel strength measurements, the compositions in Table 1 were also prepared without PEGDA. The solutions were then poured into a 100 × 15 mm Petri dish and subjected to irradiation using an E-beam system (Mevex, Stittsville, ON, Canada) at a dose of 20 kGy, as depicted in Figure 2. To determine the optimal irradiation dose for hydrogel production, we performed E-beam irradiation on an aqueous AMPS solution in the range of 5–60 kGy. Lower doses resulted in gel formation, but the gel fraction was insufficient, while too high doses resulted in reduced gel fraction due to chain scission reactions. After a comprehensive evaluation of the results, we selected 20 kGy as the optimal irradiation dose. An electron beam of 10 MeV and 8 kW was used, and the under-beam conveyor (UBC) speed was 0.648 m/min. Considering the UBC, the irradiation time per Petri dish (100 × 15 mm) was approximately 13.89 s. The pH of the final hydrogel solution was approximately 6.5, and the thickness of the cured hydrogel was approximately 3 mm. The non-volatile residue was approximately 40%.

### 2.3. Measurements

The Fourier-transform infrared spectroscopy (FT-IR) spectra of the TA and dried hydrogel were obtained by preparing a KBr pellet (sample:KBr = 1:100). The dry hydrogel was prepared as follows: 1 g of hydrogel was placed in a beaker with a sufficient amount of distilled water and stirred at room temperature for 24 h. Afterward, it was filtered, and the obtained gel was dried in a vacuum oven at 60 °C for 24 h. Measurements were performed in transmittance mode with a scan range of 4000–600 cm^−1^, 32 scans and a resolution of 4 cm^−1^.

The degree of grafting was determined by analysis of residual TA by extraction. A dried hydrogel sample weighing 0.1 g was then wrapped in metal paper (20 mesh) and extracted in distilled water for 24 h at room temperature. The extracted hydrogel was dried in a vacuum oven (60 °C, 24 h). To maximize the extraction of TA, this process was repeated three times. After removing the water from the extracted solution, a sample was prepared by dissolving the residue in 10 g of water. The analysis was performed using a 1260 Infinity II Prime LC system (Agilent, Santa Clara, CA, USA). The high-performance liquid chromatography (HPLC) measurement conditions included a mobile phase composed of methanol: 1% acetic acid (in water) with a ratio of 6:4. Ultraviolet (UV) detection was performed at a wavelength of 280 nm, and a symmetric C18 column (4.6 × 250 mm, 5 µm) was used. 

The gel fraction of the hydrogel was obtained through extraction, and the detailed procedure was as follows: The hydrogel was dried in a vacuum oven (60 °C, 24 h). The dried gel (0.1 g) was then wrapped in metallic paper (20 mesh) and extracted in distilled water for 24 h at room temperature. The extracted gel was subjected to drying in a vacuum oven (60 °C, 24 h). Afterward, the dried gel was weighed (w_2_). The gel fraction was determined using the following equation:

(1) $$Gel\;fraction\;\left(\%\right)=\frac{w_{2}}{w_{1}}\times 100$$

where *w*_1_ is the weight of the dried hydrogel, and *w*_2_ is the weight of the dried sample after extraction. At least 3 samples were used for this measurement. 

To measure the swelling ratio, a dried hydrogel (square form, 2 g) was immersed in a significant amount of distilled water at room temperature. At specified time intervals, the hydrogel was removed from the water, gently wiped on the surface with a tissue, and weighed. The swelling ratio was then calculated using the following equation:

(2) $$Swelling\;ratio\;\left(\%\right)=\frac{w_{t}-w_{0}}{w_{0}}\times 100,$$

In the provided equation, *w_t_* represents the weight of the hydrogel after it has swelled for a specific time, and *w*_0_ represents the initial weight of the dried hydrogel.

The viscoelastic properties of the hydrogel were determined using an MCR 102 rheometer (Anton Paar, Graz, Austria). A circular hydrogel sample (diameter: 25 mm and thickness: 3 ± 0.3 mm) was placed in the center of the lower plate. After the parallel plate was set up, each sample underwent testing in frequency sweep mode. The frequency range for the sweep was set to range from 0.1 to 10 Hz while maintaining a constant applied strain of 0.1%.

Scanning electron microscopy (SEM) was employed to examine the porous structure of the hydrogels. The hydrogel samples were refrigerated at −20 °C for 48 h and then lyophilized at −50 °C for 72 h using an FDU-1200 lyophilizer (EYELA, Tokyo, Japan). After the freeze-drying process, the dried hydrogel was sputter-coated for a duration of 30 s using a 108auto Sputter Coater (Cressington Scientific Instruments, Watford, UK). The coated sample was then characterized using a JSM-7601F SEM (JEOL, Tokyo, Japan).

The peel strength (90 degree) of the hydrogels was measured using a peel tester (SurTA 2D, Suwon, Republic of Korea). Prior to measurement, the substrates (stainless steel 304, glass, polypropylene, wood, pork skin) were cleaned with acetone. In the case of pork skin, the frozen sample was thawed in a refrigerator (4 °C) for 24 h, and the surface was cleaned with water and acetone to ensure cleanliness prior to use. The cut hydrogel samples (20 × 60 mm rectangles with 3 ± 0.3 mm thickness) were bonded to kraft paper (backing). They were then applied to a substrate and left for 20 min. The test was subsequently conducted, measuring a minimum of 5 samples, with a speed set at 5 mm/s.

Tensile tests were carried out using a peel tester (SurTA 2D, Republic of Korea). The hydrogel samples were formed into dog bone-shaped specimens according to the guidelines of ASTM D638 (Type V) [22]. Each specimen had an overall length of 3.18 mm, a gauge length (I0) of 9.53 mm, and a thickness of 3 ± 0.3 mm. Tests were conducted on a minimum of 3 specimens at a constant test speed of 100 mm/min.

Electrochemical impedance spectroscopy (EIS) was used to measure the ionic conductivity of the AMPS/TA hydrogels. An Autolab PGSTAT 204 potentiostat (Metrohm AG, Herisau, Switzerland) was employed to determine the ionic conductivities of the hydrogels. The hydrogel samples were prepared as squares (10 × 10 mm) and sandwiched between two indium tin oxide (ITO) glass plates (20 × 20 mm). The measurement was performed under open-circuit conditions, 0.1 Hz to 100 kHz frequency range, and 10 mV excitation voltage. The impedance data were analyzed and fitted using the NOVA 2.1.4 software provided by Metrohm Autolab B.V. The bulk resistance (*R*_0_) was determined as the x-intercept in the high frequency region [23,24]. The calculated values of Ro were subsequently employed in the calculation of ionic conductivities for the hydrogels, utilizing the following equation:

(3) $$\sigma =\frac{l}{R_{0}\times A}$$

In the equation, *l* is the thickness, *R_0_* is the bulk resistivity, *A* is the area, and *σ* is the ionic conductivity of the hydrogel. Measurements were taken at least 3 times, and the average was used for analysis and calculations.

The OWON B35T digital multimeter (OWON, Zhangzhou, China) was used to record the changes in real-time relative resistance in the hydrogel under a specific strain. To measure the resistance change, the hydrogel sample (10 × 60 × 3 mm) was wrapped with copper wire and attached to the index finger and then connected to the multimeter. The relative resistance change was then calculated using the following equation:

(4) $$\frac{∆R}{R_{0}}(\%)=\frac{\left(R-R_{0}\right)}{R_{0}}\times 100$$

In the provided equation, *R* is the stretched resistance of the hydrogel, *R_0_* is the original resistance of the hydrogel.

## 3. Results and Discussion

### 3.1. Characterization of AMPS/TA Hydrogels Prepared Using E-Beam Irradiation

**Figure 3:**
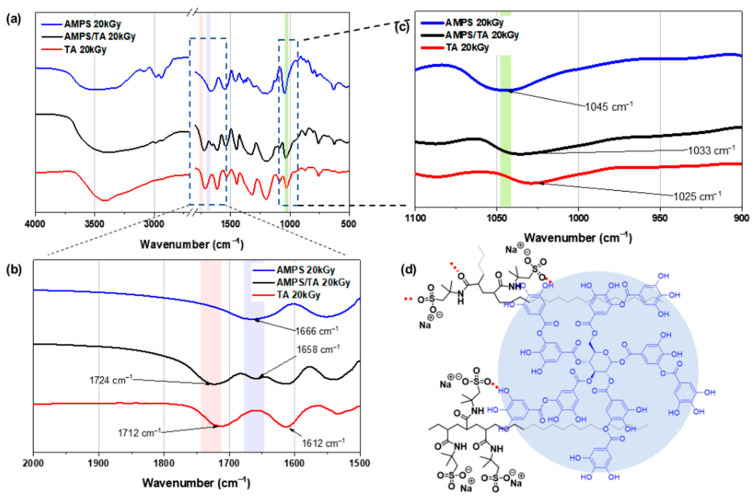
FTIR spectra of AMPS and AMPS/TA hydrogels, and TA: (**a**) FTIR spectra of AMPS and AMPS/TA hydrogels obtained after irradiation of 20 kGy E-beam and subsequent extraction, (**b**) FTIR spectra in the range of 2000 and 1500 cm^−1^, (**c**) FTIR spectra in the range of 1100 and 900 cm^−1^, and (**d**) schematic diagram depicting the formation of H-bonds between AMPS polymer and TA.

AMPS/TA hydrogels were successfully synthesized using a simple one-pot process after E-beam irradiation. However, it was very difficult to determine their exact structure. So, we decided to use indirect methods such as extraction techniques to better understand their structure. To confirm the formation of covalent bonds between the AMPS polymer chain and TA by E-beam irradiation, the hydrogels were extracted with distilled water, followed by thorough drying in a vacuum oven according to the procedure described in Section 2.3. Figure 3a–c presents the FT-IR spectra acquired after the extraction process, representing the AMPS and AMPS/TA hydrogels prepared by 20 kGy E-beam irradiation. 

In Figure 3a, the presence of aromatic C=O (1712 cm^−1^) and aromatic C–O (1612 cm^−1^) peaks in both TA and the AMPS/TA sample indicates the formation of covalent bonds between TA and AMPS polymer. Figure 3b shows the carbonyl stretching peak at 1666 cm^−1^ corresponding to the amide of AMPS in AMPS/TA (TA9) complex. This peak undergoes a shift to a lower wavenumber (1658 cm^−1^) due to the formation of H-bonds between C=O and OH groups. Moreover, the C=O vibration of TA exhibits a shift from 1712 to 1724 cm^−1^, which indicates that the vibration energy of the C=O bonding is strengthened and influenced by the hydrogen donor effect [8,25]. Furthermore, in Figure 3c, the –S=O stretching peak of sulfate in AMPS is observed at 1045 cm^−1^, while in the complex it is observed at 1033 cm^−1^, which forms H-bonds with the OH groups of TA. The interaction of AMPS polymer and TA is determined by the type of interaction (H-bond or ionic bond, depending on the chemical structure), the concentration of polymer, TA, and pH [8].

**Figure 4:**
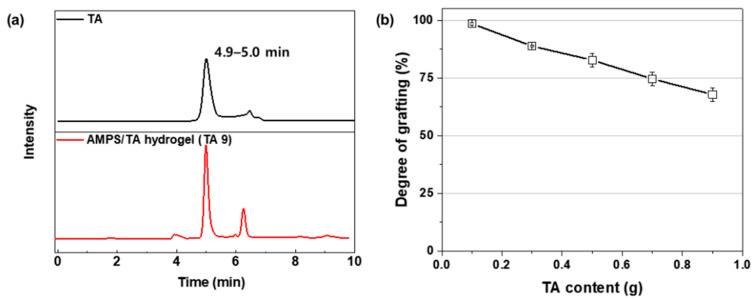
Chromatographic analysis of TA and the extracted solution of AMPS/TA hydrogel: (**a**) Chromatograms of TA and the extracted solution, and (**b**) degree of grafting for TA plotted as a function of the added mass.

The formation of covalent bonds in the AMPS/TA hydrogel, achieved through E-beam irradiation, was confirmed by IR analysis. However, the determination of grafting extent was challenging and required further investigation via HPLC analysis of the extracted solution. Figure 4a,b shows the chromatograms of TA and the extracted solution and the degree of grafting for TA. The unreacted TA content ranged from 2 to 297 mg depending on the amount added, and the degree of grafting was calculated based on this value and the mass added. The results revealed a decrease in the degree of grafting with increasing TA content, indicating that grafting with TA occurs with a concomitant radical scavenging effect. 

**Figure 5:**
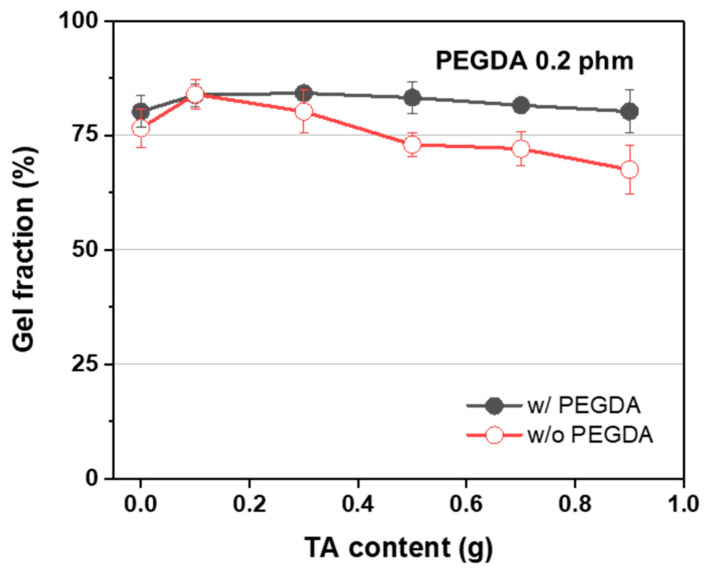
Relationship between the TA content and the gel fraction of AMPS/TA hydrogels with and without PEGDA (0.2 phm).

Figure 5 showcases the gel fraction analysis of AMPS/TA hydrogels with and without the presence of PEGDA, a crosslinker. The gel fraction of AMPS/TA hydrogels without PEGDA exhibited a slight increase from 76.6% (TA0) to 84.0% (TA1), followed by a decrease to 67.5% (TA9). This trend suggests that the addition of a small amount of TA (0.1 g) results in an increased gel fraction due to crosslinking facilitated by TA grafting. However, as the amount of TA added continues to increase, the gel fraction appears to decrease, likely influenced by the radical scavenging effect of TA. Conversely, when PEGDA, the crosslinker, was introduced, the gel fraction remained at 80.2% even with 0.9 g of TA. This observation suggests that the gel fraction is influenced by the presence of diacrylate as a crosslinker.

**Figure 6:**
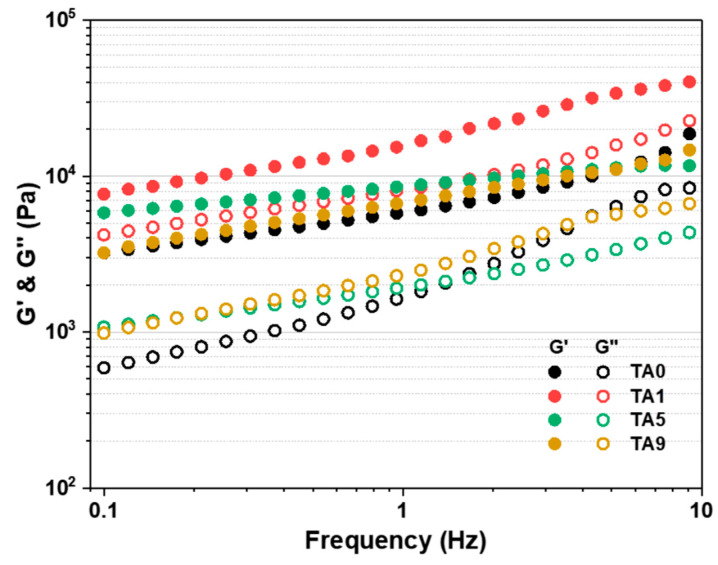
Frequency-dependent storage and loss moduli of AMPS/TA hydrogels with PEGDA (0.2 phm).

The storage modulus of a hydrogel is closely related to its cohesion, and the small amplitude oscillatory shear (SAOS) method is widely accepted as a suitable approach to determine the storage modulus of swollen hydrogels. Figure 6 presents the results of rheometer measurements performed on the hydrogels over a frequency range at room temperature. Reproducible results were not achieved with AMPS/TA hydrogels without PEGDA, primarily due to challenges with precise deformation resulting from the inherent softness and strong adhesion of the hydrogels. Consequently, our evaluation focused exclusively on samples containing 0.2 phm PEGDA.

For all hydrogels, an increase in storage modulus with increasing frequency (i.e., shorter relaxation time) was observed, indicating that at higher frequencies, the polymer chains do not have sufficient time to relax, resulting in decreased flexibility and increased stiffness. In addition, the storage modulus (G′) exceeded the loss modulus (G″) in all frequency ranges, indicating the predominance of elastic over viscous behavior and highlighting the mechanical stiffness of the hydrogels. In particular, the incorporation of small amounts of TA (0.1 g) resulted in a significant increase in the storage modulus over all frequency ranges. However, as the TA content increased, both G′ and G″ showed a decrease. This observation can be attributed to the beneficial grafting effect of adding a small amount of TA, which improves hydrogel cohesion. Conversely, higher TA content enhances the radical scavenging effect, resulting in an increased presence of ungrafted TA, which acts as a form of plasticizer, facilitating polymer chain relaxation.

### 3.2. Adhesion and Tensile Properties of AMPS/TA Hydrogels

**Figure 7:**
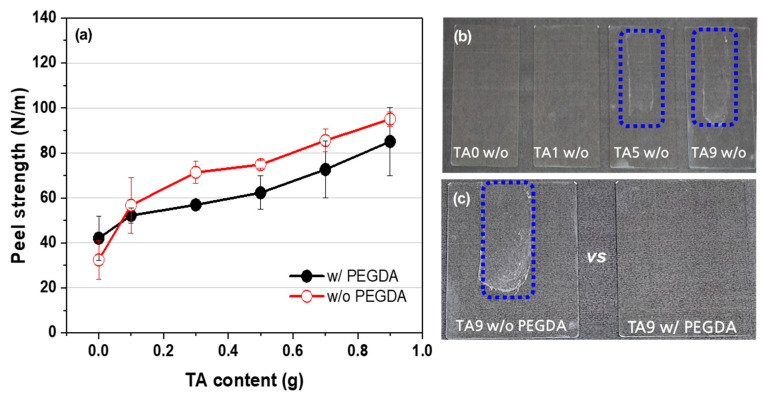
Peel strength analysis of AMPS/TA hydrogel on a glass substrate: (**a**) 90° peel strength measurement, (**b**) visual inspection of residue (inside the blue rectangle) from AMPS/TA hydrogel without PEGDA, and (**c**) comparison of residue (inside the blue rectangle) formation between AMPS/TA hydrogels with and without PEGDA.

The peel strength of the AMPS/TA hydrogel prepared by E-beam irradiation was evaluated on a glass substrate as shown in Figure 7a. The peel strength exhibited an upward trend with increasing TA content, both in the absence and presence of PEGDA. The incorporation of TA in hydrogels is known to enhance adhesion through the involvement of numerous hydroxyl groups (catechol, pyrogallol) that form H-bonds with the substrates [6,7]. In the absence of PEGDA, the peel strength showed a substantial increase due to the soft nature of the hydrogel, which facilitated easy contact with the substrate. However, when the TA content exceeded 0.5 g, cohesive failure occurred, and residues of the hydrogel were observed on the substrate surface (Figure 7b). Conversely, when 0.2 phm of PEGDA was added, no residue was observed on the substrate surface even when 0.9 g of TA was included (Figure 7c).

**Figure 8:**
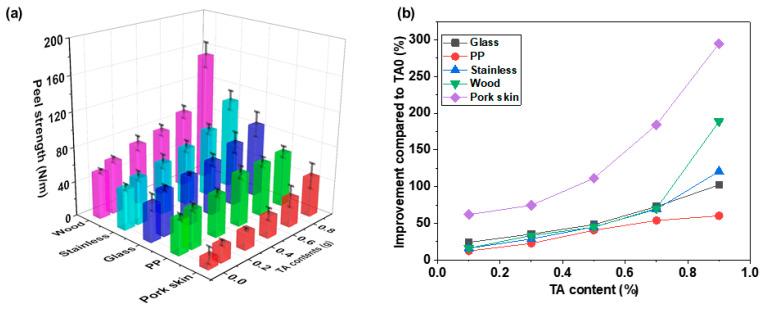
Peel strength variation of AMPS/TA hydrogels incorporating 0.2 phm PEGDA on different substrates: (**a**) Effect of TA content and substrate type on 90° peel strength, and (**b**) percentage increase in peel strength.

Figure 8a shows the 90° peel strength results for AMPS/TA hydrogels with PEGDA. Additionally, Figure 8b has been included to provide further insight into the data from Figure 8a by illustrating the degree of improvement in peel strength compared to TA0. The adhesion of the hydrogels showed an increase with the addition of TA across all substrate types. TA possesses an –OH group, which is known to form reversible non-covalent or irreversible covalent interactions with various organic and inorganic substrates, including wood, glass, and metal, resulting in enhanced adhesion [26,27,28]. Evaluation of the TA9 samples revealed a descending order of adhesion: wood exhibited the highest adhesion, followed by SUS, glass, PP, and pork skin. The effect of TA addition on adhesion enhancement was comparatively less pronounced on the non-polar PP substrate (TA0: 39.4 N/m, TA9: 62.5 N/m). However, on the wood substrate, specific interactions such as π–π interaction and hydrogen bonding led to a substantial increase in adhesion of about 180%, escalating from 52.5 N/m to 151.6 N/m. On the other hand, although the adhesion value for pork skin was relatively low, the addition of TA resulted in the most significant increase in adhesion, approximately 300%. This significant improvement in adhesion is attributed to the increased presence of phenolic groups within the AMPS/TA hydrogel network. The phenolic groups of TA facilitate a dynamic Schiff base binding reaction with the amino groups in the skin tissue, resulting in high interfacial adhesion [29].

### 3.3. Mechanical Properties of AMPS/TA Hydrogels

The tensile test results for AMPS/TA hydrogel are shown in Figure 8. The tensile stress showed a significant increase at TA1, followed by a subsequent decrease with increasing TA content, resulting in lower stress values compared to TA0 starting from TA5. Elongation showed a slight increase with the addition of TA and then remained at similar values.

**Figure 9:**
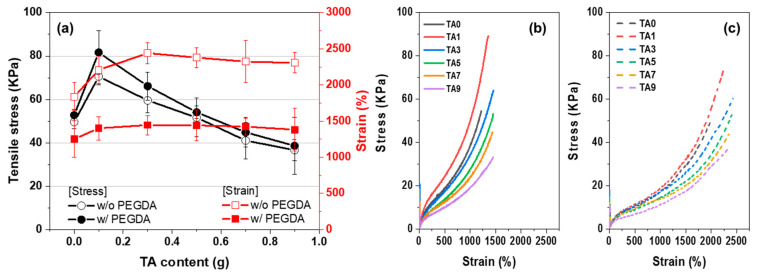
Tensile properties of AMPS/TA hydrogels: (**a**) Influence of TA content on tensile test results, (**b**) stress–strain behavior with PEGDA, and (**c**) stress–strain behavior without PEGDA.

Considering the combined analysis of Figure 6 and Figure 9, it can be concluded that the hydrogel network is strengthened by the grafting reaction up to TA5. However, beyond this threshold, the radical scavenging effect of TA leads to an accumulation of ungrafted free TA. Consequently, the plasticizing effect of this ungrafted TA contributes to a reduction in tensile stress and an increase in strain.

### 3.4. Ionic Conductivity and Swelling Properties of AMPS/TA Hydrogels

**Figure 10:**
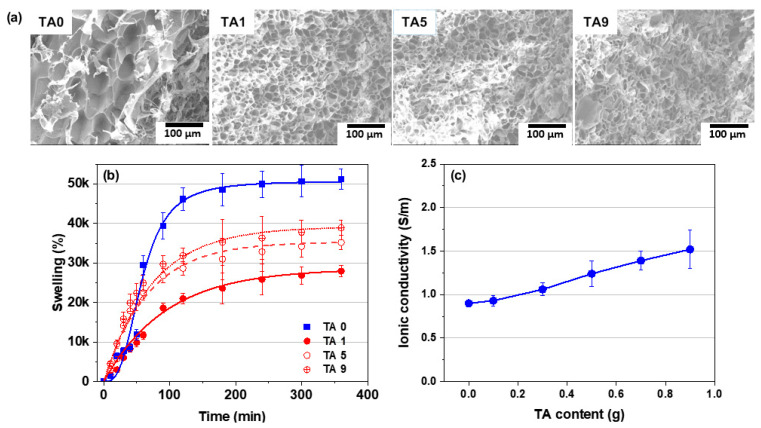
(**a**) SEM images of cross-section of AMPS/TA hydrogels containing 0.2 phm PEGDA after lyophilization (100× magnification), (**b**) swelling ratio analysis, and (**c**) ionic conductivity evaluation.

Figure 10a shows the changes in the pore structure of the hydrogel as a function of TA content. The addition of TA resulted in a significant reduction in pore size, while the subsequent changes in pore size with increasing TA content were relatively small. These results are consistent with the results shown in Figure 6, where the introduction of TA resulted in a denser network structure, which contributed to a decrease in water absorption (Figure 10b). The less pronounced swelling observed in the denser networks is attributed to constrained stretching, resulting from reduced network elasticity due to a higher density of crosslinking points.

Figure 10c shows the influence of TA content on the ionic conductivity of the hydrogel. There is a noticeable trend of increasing ionic conductivity with higher TA content. It is well known that TA has the ability to bind with metal ions, acting as ionic bridges and allowing the preparation of hydrogels with favorable conductivity [30,31,32]. Consequently, the observed increase in ionic conductivity with increasing TA content is attributed to the increased presence of ionic bridges in the prepared hydrogels, which improves the overall ionic conductivity performance.

**Figure 11:**
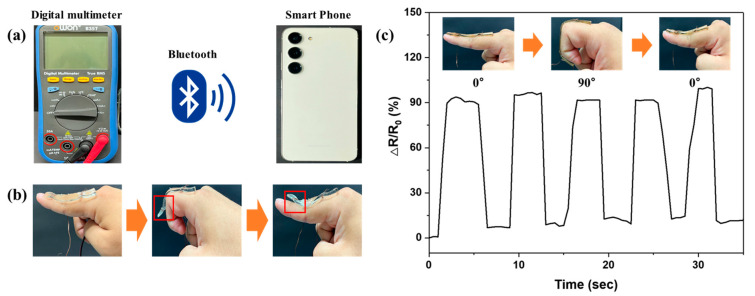
(**a**) Real-time detection of human motion using Bluetooth device. (**b**) Failed to measure due to poor skin adherence of TA0. (**c**) Real-time relative resistance response of TA9 by finger bending.

The wireless human body motion detection experiment was conducted using the Bluetooth function of a digital multimeter (Figure 11a). Figure 11b shows the finger bending motion of the TA0 hydrogel. However, due to the poor skin adhesion of the TA0 hydrogel, accurate measurements were not possible. In contrast, the TA9 sample, which has excellent skin adhesion, allowed for real-time measurements of relative resistance, as shown in Figure 11c. As the finger bent, the relative resistance of the hydrogel sensor increased, exhibiting a regular pattern. This is because the increased resistance was a result of the narrowed porosity of the stretched hydrogel microstructure as the finger bent [33]. After the finger returned to its original position, there was little change in the relative resistance, demonstrating that the TA9 hydrogel can be used as a hydrogel strain sensor to accurately detect bending motion [34].

## 4. Conclusions

In this study, we successfully prepared an ionically conductive hydrogel by subjecting an aqueous solution of AMPS and TA to E-beam irradiation for a short time (within 15 s) at room temperature.

Unlike conventional thermal or UV-induced radical polymerization methods, E-beam irradiation demonstrated the grafting of TA onto the AMPS polymer chain. The degree of TA grafting showed a linear decrease with increasing TA content. Furthermore, the added TA was found to form hydrogen bonds with the amide and sulfonate groups of the AMPS polymer chain. As the TA content increased, the gel fraction of the AMPS/TA hydrogels decreased. Rheometer measurements revealed that the increased presence of ungrafted TA facilitated the relaxation of the AMPS polymer chain, resulting in a decrease in the tensile stress of the hydrogels.

The peel strength of the AMPS/TA hydrogels showed an increase with higher TA content, which was attributed to improved contact with the substrate due to the decreased modulus. In addition, the peel strength was strongly influenced by the presence or absence of specific interactions between TA and the substrate. In particular, the wood substrate exhibited the highest peel strength, while the greatest increase in peel strength was observed with pork skin. Conversely, the ionic conductivity of the AMPS/TA hydrogels showed an almost linear increase with increasing TA content.

In conclusion, we successfully used E-beam irradiation to prepare hydrogels with remarkable peel strength and ionic conductivity at room temperature. This method offers advantages over conventional UV irradiation, allowing for easy preparation of hydrogels with high peel strength and ionic conductivity by incorporating TA and ionic monomers with radical scavenging effects.

## Figures and Tables

**Table 1:** Detailed composition of the AMPS/TA hydrogel.

Sample Code	AMPS Salt Solution (g)	H_2_O (g)	TA		PEGDA	
			(g)	(phm *)	(g)	(phm *)
**TA0**	80 (40/40)	20	-	-	0.08	0.2
**TA1**		20.1	0.1	0.25		
**TA3**		20.3	0.3	0.75		
**TA5**		20.5	0.5	1.25		
**TA7**		20.7	0.7	1.75		
**TA9**		20.9	0.9	2.25		

* phm: parts per hundred monomer (AMPS).

## Data Availability

The data presented in this study are available on request from the corresponding author.
